# Clinical Spectrum and One-Year Outcomes of Central Nervous System Vasculitis Secondary to Systemic Autoimmune Disorders: A Retrospective Cohort Study

**DOI:** 10.7759/cureus.95050

**Published:** 2025-10-21

**Authors:** Shahul Irfan, Vishwa Venkatesh, Soorya Senthilkumar, Aarushi H Sevak, V Poshak Navaneeth Reddy, Harikrishnan Parthipan, Shreenaa Sundararajan, Priyadharshini Shanmugam, Harini Ravichandran, Sadaf Sultana S Doctor

**Affiliations:** 1 Internal Medicine, Periyar Government Hospital, Chennai, IND; 2 Medicine, ESIC (Employees' State Insurance Corporation) Medical College and Post Graduate Institute of Medical Science and Research, Chennai, IND; 3 General Medicine, Warrington Hospital, Warrington, GBR; 4 Medical School, Caucasus University, Tbilisi, GEO; 5 Medical School, Sri Ramachandra Institute of Higher Education and Research, Chennai, IND; 6 Geriatrics, Langbank Medical Center, Liverpool, GBR; 7 Paediatrics, Torbay and South Devon NHS foundation Trust, Torquay, GBR; 8 General Medicine, Kauvery Hospital, Chennai, IND; 9 Research, ESIC (Employees' State Insurance Corporation) Medical College and Post Graduate Institute of Medical Science and Research, Chennai, IND; 10 Internal Medicine, Dr. Kiran C. Patel Medical College and Research Institute, Bharuch, IND

**Keywords:** auto-immune condition, cns vasculitis, mri brain, seizure, stroke

## Abstract

Introduction

Central nervous system (CNS) vasculitis (CNSV) is a rare but serious complication of systemic autoimmune disorders, including systemic lupus erythematosus, antineutrophil cytoplasmic antibodies (ANCA)-associated vasculitis, rheumatoid arthritis, polyarteritis nodosa, Sjögren’s syndrome, and mixed connective tissue disease. Existing literature predominantly addresses primary CNSV, with limited systematic follow-up of secondary CNS involvement. This study aimed to describe the clinical spectrum, laboratory and imaging features, treatment strategies, and one-year outcomes of CNSV secondary to autoimmune diseases, while identifying predictors of prognosis.

Methods

This retrospective cohort study was conducted at Madras Medical College, Chennai, India, between May 2024 and May 2025. Consecutive adult patients with established systemic autoimmune disorders who developed CNS manifestations consistent with vasculitis were included. Patients with primary CNSV or infectious, neoplastic, or metabolic mimics were excluded. Clinical features, laboratory data, and neuroimaging findings were extracted from records using a structured proforma. Treatments were categorized as corticosteroids alone, steroids plus conventional immunosuppressants, or steroids plus biologics. Patients were followed for 12 months, with functional outcome assessed by modified Rankin Scale (mRS); a favorable outcome was defined as mRS 0-2. Relapse was defined as a new deficit after stabilization with radiological confirmation.

Results

A total of 150 patients were studied (mean age 42.6 ± 12.4 years; 54.7% female). The most common underlying disease was lupus (70 patients, 46.7%), followed by ANCA-vasculitis (35 patients, 23.3%). Stroke-like presentations were seen in 70 patients (46.7%), seizures in 40 patients (26.7%), encephalopathy in 30 patients (20%), and focal motor/sensory deficits in 50 patients (33.3%), with 65 patients (43.3%) showing overlapping features. ANA was positive in 75 patients (50%), anti-dsDNA in 55 patients (36.7%), ANCA in 45 patients (30%), and multiple antibody positivity in 50 (33.3%). Very high antibody titers and elevated C-reactive protein (CRP) correlated with poor outcomes, while moderate titers did not. MRI abnormalities were present in 135 patients (90%), most often multiple-territory infarcts in 55 patients (36.7%), lacunar infarcts in 40 patients (26.7%), and white matter hyperintensities in 45 patients (30%). At one year, favorable outcomes increased from 70 patients (46.7%) at discharge to 85 patients (56.7%); relapse occurred in 30 patients (20%), recurrent admissions in 25 patients (16.7%), and mortality was seen in 20 patients (13.3%).

Conclusion

CNSV secondary to systemic autoimmune disease is clinically heterogeneous, with frequent systemic and neurological overlap, high inflammatory burden, and multifocal imaging abnormalities. Prognosis was significantly influenced by treatment timing, inflammatory markers, and antibody titers. Early initiation of immunosuppression and combination regimens were associated with better recovery, while delayed therapy and multiple antibody positivity predicted relapse and disability. Despite adherence to guideline-based therapy, mortality and relapse remained considerable, emphasizing the need for early recognition, aggressive therapy, and structured long-term follow-up.

## Introduction

Central nervous system (CNS) vasculitis (CNSV) refers to inflammation of the blood vessels of the brain, meninges, or spinal cord, and can occur either in isolation (primary CNSV) or secondary to systemic autoimmune disorders. In secondary CNSV, systemic autoimmunity such as lupus, antineutrophil cytoplasmic antibodies (ANCA)-associated vasculitis, or rheumatoid arthritis leads to vascular inflammation in the CNS, causing ischemic or hemorrhagic injury, seizures, encephalopathy, and other neurological deficits. This disorder is relatively rare but carries high morbidity and risk of permanent disability if diagnosis and treatment are delayed. Studies emphasize that distinguishing CNSV from its many mimics, including infectious, neoplastic, metabolic, or non-inflammatory vascular conditions, is especially challenging [1]. A particular issue is diagnostic delay. Because symptoms are often non-specific, such as headache, cognitive changes, or focal neurological deficits, and because available diagnostic modalities have imperfect sensitivity and specificity, many patients experience a prolonged interval before the correct diagnosis is established. Delayed diagnosis has been consistently associated with worse outcomes in systemic vasculitides [2]. Moreover, even once treatment is initiated, relapses are common, and long-term functional outcome may remain suboptimal unless aggressive and sustained immunosuppressive therapy is used [3].

Despite existing literature on primary CNSV and case reports of secondary CNSV, there remains a paucity of structured follow-up studies focusing on patients with CNSV secondary to systemic autoimmune diseases, particularly in resource-limited settings. It is not well known which clinical, imaging, or treatment variables at presentation are predictive of favorable one-year outcomes, such as functional status, relapse, and mortality [4]. The novelty of the present study lies in examining a defined cohort of secondary CNSV patients, with one-year follow-up, and focusing on a limited number of high-yield predictors rather than broad associations. For the purpose of this study, secondary CNSV was defined as CNS involvement occurring in patients with an established systemic autoimmune disease, presenting with compatible neurological manifestations, and supported by characteristic neuroimaging and/or cerebrospinal fluid findings after exclusion of infectious, neoplastic, and metabolic mimics. Accordingly, our primary aim was to assess functional status at one year in patients with CNSV secondary to systemic autoimmune disorders, and to identify early predictors of favorable versus poor outcomes. The primary objective was to determine the proportion of patients achieving a favorable functional outcome (modified Rankin Scale 0-2) at 12 months. Secondary objectives included: (i) estimation of the relapse rate within 12 months; (ii) comparison of outcomes across initial treatment strategies (steroids alone versus combination with immunosuppressants or biologics); and (iii) assessment of the effect of diagnostic delay, defined as time from neurological symptom onset to immunosuppressive treatment, on one-year functional outcome and mortality. The study design, data collection, and reporting were conducted in accordance with the STROBE (Strengthening the Reporting of Observational Studies in Epidemiology) guidelines for observational cohort studies.

## Materials and methods

This retrospective cohort study was conducted in the Department of Neurology at Madras Medical College, Chennai, India, over a one-year period from May 2024 to May 2025, after obtaining approval from the Institutional Human Ethics Committee, Madras Medical College (approval number: NEW/EC/MMC/2319). The study design, data collection, and reporting were conducted in accordance with the STROBE (Strengthening the Reporting of Observational Studies in Epidemiology) guidelines for observational cohort studies.

Eligibility criteria

All consecutive patients aged 18 years or above with a confirmed systemic autoimmune disease, diagnosed in accordance with the respective American College of Rheumatology/European League Against Rheumatism (ACR/EULAR) classification criteria [5], who developed central nervous system manifestations during the study period were screened for inclusion. Patients were eligible if they presented with neurological features consistent with vasculitic involvement of the central nervous system, such as stroke-like episodes, seizures, encephalopathy, or other focal neurological deficits, and if radiological investigations with magnetic resonance imaging (MRI) or magnetic resonance angiography (MRA) modalities suggested an underlying vasculitic process. Patients were excluded if they had primary angiitis of the CNS without systemic autoimmune disease, if they had infectious, neoplastic, or metabolic disorders capable of mimicking vasculitis, such as tuberculous meningitis, viral encephalitis, lymphoma, or reversible cerebral vasoconstriction syndrome, or if they had incomplete clinical records or were lost to follow-up before the one-year evaluation. In addition, comorbid vascular risk factors, including hypertension, diabetes mellitus, dyslipidemia, ischemic heart disease, and smoking history, were recorded to identify potential confounders and to exclude patients whose cerebrovascular events were more consistent with non-vasculitic etiologies.

Sample size

The sample size was calculated using a single-proportion formula, assuming a 10% prevalence of CNS involvement among patients with systemic autoimmune disorders, with a 95% confidence level and 5% absolute precision, which yielded a minimum requirement of 139 patients. A total of 150 eligible patients were included in the study, thereby fulfilling the required sample size.

Data collection and procedures

All data were retrieved from electronic hospital records, case files, laboratory archives, and departmental imaging repositories. The investigator independently extracted information using a pre-designed structured proforma, and discrepancies were resolved through consensus with a senior consultant neurologist. Demographic data included age, sex, occupation, and socioeconomic background. Disease-related data comprised type and duration of systemic autoimmune disorder, previous treatment received for the primary autoimmune disease, and extent of extra-neurological systemic involvement such as renal, pulmonary, cardiac, dermatological, or musculoskeletal features. Neurological presentation was documented in detail, specifying whether it was ischemic or hemorrhagic stroke, seizure at onset, acute confusional state or encephalopathy, focal deficits, headache, or progressive cognitive decline. The temporal profile of symptoms, the presence of prodromal systemic flares, and the duration between onset of neurological complaints and first presentation to hospital were noted, with the last used to compute diagnostic delay. Cerebrospinal fluid (CSF) analysis was performed in patients where the clinical presentation raised the possibility of infectious, demyelinating, or neoplastic mimics, and included assessment of protein, glucose, cytology, and oligoclonal bands.

Laboratory evaluation included both routine and disease-specific investigations. Routine tests comprised complete blood counts, renal and liver function tests, electrolytes, and coagulation profile. Inflammatory markers included erythrocyte sedimentation rate (Westergren method) and C-reactive protein (CRP) (immunoturbidimetry). Autoantibody profiling was performed according to standardized hospital protocols: antinuclear antibody (ANA) and anti-dsDNA by enzyme-linked immunosorbent assay (ELISA) and immunofluorescence, where required, ANCA testing (MPO and PR3) by ELISA, complement fractions C3 and C4 by nephelometry, and antiphospholipid antibodies, including lupus anticoagulant and anticardiolipin antibodies, using coagulation and immunoassay platforms.

Autoantibody titers were classified into three categories based on established laboratory thresholds and prior cohort definitions. ANA was considered normal at <1:80, high at 1:160-1:320, and very high at ≥1:640 by immunofluorescence. Anti-double-stranded DNA (anti-dsDNA) titers were defined as normal <30 IU/mL, high 30-100 IU/mL, and very high >100 IU/mL by ELISA. Anti-neutrophil cytoplasmic antibody (ANCA, MPO/PR3) was defined as normal <20 U/mL, high 20-50 U/mL, and very high >50 U/mL. Anti-cyclic citrullinated peptide antibody (anti-CCP), second-generation assay, was classified as normal <20 U/mL, high 20-60 U/mL, and very high >60 U/mL. Rheumatoid factor (RF) was considered normal <20 IU/mL, high 20-60 IU/mL, and very high >60 IU/mL. For analytic purposes, both the effect of high/very high titers and the presence of multiple antibody positivity (≥2 markers) were examined for their association with clinical outcomes. Where indicated, CSF analysis was carried out under aseptic precautions, measuring opening pressure, protein, glucose, cell counts, and cytology, with additional studies such as oligoclonal bands and infectious panels if clinically required, primarily to exclude mimicking conditions. All laboratory samples were processed in the central laboratory of Madras Medical College immediately upon collection, using standardized processing protocols and external quality assurance schemes to ensure reproducibility of results.

For patients fulfilling the inclusion criteria with neurological manifestations suggestive of CNSV, neuroimaging formed a cornerstone of case ascertainment. MRI brain was performed on 1.5 Tesla or 3 Tesla machines using a standardized protocol including axial and coronal T1-weighted, T2-weighted, fluid-attenuated inversion recovery (FLAIR), diffusion-weighted imaging (DWI), apparent diffusion coefficient (ADC) maps, gradient echo or susceptibility-weighted imaging, and post-contrast T1 sequences. MRA, typically time-of-flight MRA, was carried out to assess large and medium vessel involvement, and vessel wall imaging was performed to detect concentric characteristics of vasculitis. Infarcts were classified as small-vessel (lacunar or cortical microinfarcts) or large-vessel (territorial or cortical-subcortical). Hemorrhages, microbleeds, and areas of leukoencephalopathy were also noted. Lacunar infarcts were defined as ≤15 mm lesions on DWI/ADC; white matter hyperintensities were identified on FLAIR sequences and graded using the Fazekas visual scale, where applicable. Vessel wall enhancement was considered vasculitic when showing concentric mural enhancement with or without luminal stenosis

Treatment details were extracted from inpatient charts and follow-up notes, and categorized into three groups: corticosteroids alone, corticosteroids plus conventional immunosuppressants such as cyclophosphamide, azathioprine, or mycophenolate mofetil, and corticosteroids plus biologic therapy such as rituximab. All patients received high-dose intravenous methylprednisolone pulses (1 g daily for three to five days) as induction, where feasible, followed by tapering oral prednisolone. The choice of additional immunosuppression or biologic therapy was individualized based on disease severity, comorbidities, and physician discretion. Time to treatment initiation was carefully documented and dichotomized as seven days or less versus more than seven days from the onset of neurological symptoms. Supportive care, including antiepileptics for seizures, antiplatelets or anticoagulants in those with antiphospholipid antibodies, and rehabilitation measures were also recorded.

Patients were followed for 12 months from the index neurological event, either through outpatient visits or telephonic contact where necessary. Functional status was assessed at discharge, at three to six months, and at 12 months using the modified Rankin Scale [6], with a favorable functional outcome defined as a score of 0-2 and a poor outcome as 3-6. Relapse was defined as the occurrence of new neurological deficits attributable to vasculitis, arising after an initial period of clinical stabilization, and confirmed by the presence of new lesions on MRI or MRA studies. Mortality data were collected both from hospital records and by direct family contact when required. Cases with incomplete records or loss to follow-up before one year were excluded at the screening stage, and the final analysis included only patients with complete 12-month follow-up data.

Data analysis

Statistical analysis was performed using IBM SPSS Statistics for Windows, version 26.0 (IBM Corp., Armonk, New York, United States). Continuous variables were expressed as mean with standard deviation (SD) for normally distributed data or median with interquartile range (IQR) for skewed data, while categorical variables were expressed as frequency and percentage. Categorical variables were compared using the Chi-square test. Odds ratios (ORs) with 95% confidence intervals (CIs) were calculated for categorical associations, and a p-value less than 0.05 was taken as statistically significant. Multivariable logistic regression included predefined covariates: age, sex, autoimmune disease type, CRP level, antibody titer category, comorbidity burden, and MRI severity. Analyses used complete-case data without imputation as missing variables were <5%.

## Results

A total of 150 patients were included in the study. The mean age was 42.6 years (SD 12.4), and there was a slight female predominance, with 68 male and 82 female patients. The median duration of the autoimmune disease before the onset of CNS involvement was five years (IQR 3-8). Regarding the distribution of underlying autoimmune disorders, systemic lupus erythematosus was the most common (n=70, 46.7%), followed by ANCA-associated vasculitis (n=35, 23.3%), rheumatoid arthritis (n=20, 13.3%), polyarteritis nodosa (n=15, 10%), Sjögren’s syndrome (n=6, 4%), and mixed connective tissue disease (n=4, 2.7%) (Table 1).

**Table 1:** Baseline demographic and clinical characteristics (N=150) Data given as frequency (percentage) unless otherwise indicated IQR: interquartile range; ANCA: anti-neutrophilic cytoplasmic antibody

Variable	Value
Age (years), mean ± SD	42.6 ± 12.4
Sex (Male/Female), n	68/82
Duration of autoimmune disease (years), median (IQR)	5 (3–8)
Systemic lupus erythematosus	70 (46.7%)
ANCA-associated vasculitis	35 (23.3%)
Rheumatoid arthritis	20 (13.3%)
Polyarteritis nodosa	15 (10%)
Sjögren’s syndrome	6 (4%)
Mixed connective tissue disease	4 (2.7%)

Systemic organ involvement was frequent in this cohort. Renal manifestations were observed in 50 patients (33.3%), pulmonary involvement in 45 patients (30%), cardiac manifestations in 20 patients (13.3%), cutaneous lesions in 35 patients (23.3%), and musculoskeletal involvement in 25 patients (16.7%). These findings highlight that a significant proportion of patients had multi-organ involvement in addition to neurological manifestations (Table 2).

**Table 2:** Organ involvement data

Organ Involvement	Frequency (Percentage)
Renal involvement	50 (33.3%)
Pulmonary involvement	45 (30%)
Cardiac involvement	20 (13.3%)
Cutaneous involvement	35 (23.3%)
Musculoskeletal involvement	25 (16.7%)

Stroke-like presentations were the most frequent neurological manifestation, documented in 70 patients (46.7%). Seizures were observed in 40 patients (26.7%), while encephalopathy with altered sensorium occurred in 30 patients (20%). Headache was reported by 35 patients (23.3%), and progressive cognitive decline was seen in 15 patients (10%). Focal motor or sensory deficits were present in 50 patients (33.3%), cranial nerve involvement in 10 patients (6.7%), and movement disorders in five patients (3.3%). Importantly, 65 patients (43.3%) had overlapping neurological manifestations, meaning that more than one of the above features coexisted in the same individual (Table 3). This highlights that CNSV secondary to systemic autoimmune disorders often presents with complex and multifaceted neurological syndromes rather than a single isolated manifestation.

**Table 3:** Presenting neurological manifestations

Neurological Manifestation	Frequency (Percentage)
Stroke-like presentation (ischemic/hemorrhagic)	70 (46.7%)
Seizures	40 (26.7%)
Encephalopathy/altered sensorium	30 (20%)
Headache as primary symptom	35 (23.3%)
Progressive cognitive decline	15 (10%)
Focal motor or sensory deficits	50 (33.3%)
Cranial nerve involvement	10 (6.7%)
Movement disorders (tremor, chorea, Parkinsonism)	5 (3.3%)
Patients with overlapping manifestations	65 (43.3%)

ANA positivity was seen in 75 patients (50%), whereas Anti-dsDNA positivity was seen in 55 patients (36.7%), ANCA positivity in 45 patients (30%), and low C3 or C4 in 40 patients (326.7). Rheumatoid factor and anti-CCP antibodies were positive in 25 patients (16.7%) and 20 patients (13.3%), respectively. Multiple antibody (≥2 antibodies) positivity was seen in 50 patients (33.3%) (Table 4).

**Table 4:** Specific laboratory abnormality ANA: anti-nuclear antibody; dsDNA: double-stranded deoxyribonucleic acid; ANCA: anti-neutrophilic cytoplasmic antibody; MPO: myeloperoxidase; CCP: cyclic citrullinated peptide; C3, C4: complement 3, complement 4; CSF: cerebrospinal fluid

Specific Laboratory Abnormality	Frequency (Percentage)
ANA positivity	75 (50%)
Anti-dsDNA positivity	55 (36.7%)
ANCA positivity (MPO/PR3)	45 (30%)
Low complement levels (C3 and/or C4)	40 (26.7%)
Antiphospholipid antibody positivity	30 (20%)
Rheumatoid factor positivity	25 (16.7%)
Anti-CCP2 antibody positivity	20 (13.3%)
Oligoclonal bands in CSF (subset tested)	20 (13.3%)

Out of 150 patients, MRI was normal in 15 patients (10%), where the diagnosis of CNS vasculitis was supported by clinical features and laboratory findings. Among the remaining 135 patients (90%) with abnormal MRI findings, multiple-territory infarcts were the most frequent abnormality, seen in 55 patients (36.7%). Small-vessel lacunar infarcts were identified in 40 patients (26.7%), and large-vessel territorial infarcts in 30 patients (20%). Intracerebral hemorrhage was documented in 15 patients (10%), while white matter hyperintensities were observed in 45 patients (30%). Leptomeningeal or parenchymal enhancement was noted in 20 patients (13.3%), MR angiography abnormalities in 35 patients (23.3%), and vessel wall enhancement on high-resolution MRI in 25 patients (16.7%). Importantly, 80 patients (53.3%) demonstrated overlapping abnormalities, confirming that CNS vasculitis in systemic autoimmune disorders is often multifocal and radiologically heterogeneous (Table 5).

**Table 5:** Imaging and neurodiagnostic features FLAIR: fluid attenuated inversion recovery; MRA: magnetic resonance angiography

Imaging / Neurodiagnostic Feature	Frequency (Percentage)
Normal MRI findings	15 (10%)
Multiple-territory infarcts	55 (36.7%)
Large-vessel territorial infarcts	30 (20%)
Small-vessel (lacunar) infarcts	40 (26.7%)
Intracerebral hemorrhage	15 (10%)
White matter hyperintensities (FLAIR)	45 (30%)
Leptomeningeal or parenchymal enhancement	20 (13.3%)
MRA abnormalities (beading/stenosis/occlusion)	35 (23.3%)
Vessel wall enhancement on MRI	25 (16.7%)
Patients with overlapping imaging features	80 (53.3%)

All patients received corticosteroid induction, and the majority required additional immunosuppressive therapy. Among the 150 patients, 40 (26.7%) received cyclophosphamide, 25 (16.7%) received azathioprine, 20 (13.3%) received mycophenolate mofetil, and 30 (20%) were treated with biologics in addition to steroids. A smaller subset of 20 patients (13.3%) were maintained on steroids alone, while 15 patients (10%) received only supportive management due to poor performance status or treatment contraindications (Table 6).

**Table 6:** Treatment modalities used

Treatment Regimen	Frequency (Percentage)
Steroids + Cyclophosphamide	40 (26.7%)
Steroids + Azathioprine	25 (16.7%)
Steroids + Mycophenolate mofetil	20 (13.3%)
Steroids + Biologics (Rituximab/Belimumab, etc.)	30 (20%)
Steroids alone (no further escalation)	20 (13.3%)
Supportive management only	15 (10%)

Outcomes were tracked at discharge, three months, six months, and 12 months. At discharge, 70 patients (46.7%) had a favorable outcome (mRS 0-2), increasing to 85 patients (56.7%) at 12 months. Poor outcomes (mRS 3-5) decreased from 65 patients (43.3%) at discharge to 45 patients (30%) at 12 months. Cumulative mortality rose gradually from 15 patients (10%) at discharge to 17 patients (11.3%) at three months, 19 patients (12.7%) at six months, and 20 patients (13.3%) at 12 months. Relapse events accumulated over time, with 10 patients (6.7%) relapsing by three months, 20 (13.3%) by six months, and 30 (20%) by 12 months. Recurrent admissions, defined as two or more hospitalizations due to vasculitis flares, were observed in five patients (3.3%) by three months, 15 patients (10%) by six months, and 25 patients (16.7%) by 12 months. These cumulative trends highlight the progressive but variable disease course, with incremental mortality, relapses, and recurrent admissions despite therapy (Table 7).

**Table 7:** Short-term and follow-up outcomes (corrected mortality) (N=150) mRs: modified Ranking scale

Outcome Measure	At Discharge, n (%)	At 3 Months, n (%)	At 6 Months, n (%)	At 12 Months, n (%)
Favorable functional outcome (mRS 0–2)	70 (46.7%)	75 (50%)	80 (53.3%)	85 (56.7%)
Poor functional outcome (mRS 3–5)	65 (43.3%)	58 (38.7%)	51 (34%)	45 (30%)
Cumulative mortality (mRS 6)	15 (10%)	17 (11.3%)	19 (12.7%)	20 (13.3%)
Relapse of CNS vasculitis	—	10 (6.7%)	20 (13.3%)	30 (20%)
Recurrent admissions due to vasculitis flares	—	5 (3.3%)	15 (10%)	25 (16.7%)

Patients who received immunosuppressive therapy within seven days of symptom onset had significantly higher odds of achieving a favorable functional outcome (mRS 0-2) at 12 months compared to those treated after seven days. The OR for a favorable outcome in the delayed group was 0.34 (95%CI 0.16-0.72). Chi-square test yielded a value of 9.6 with a p-value of 0.002, confirming the statistical significance of this association (Table 8).

**Table 8:** Correlation analysis: time to immunosuppression vs 12-month functional outcome

Time to Immunosuppression	Favorable Outcome (mRS 0–2), n (%)	Poor Outcome (mRS 3–6), n (%)	OR (95% CI)	Chi-square	p-value
≤ 7 days (n=80)	55 (68.8%)	25 (31.2%)			
> 7 days (n=70)	30 (42.9%)	40 (57.1%)	0.34 (0.16–0.72)	9.6	0.002*

Elevated CRP levels demonstrated a significant correlation with poor outcomes at 12 months, with both high (p=0.03) and very high levels (p=0.001) showing progressively lower odds of favorable recovery compared to normal values. For autoantibody titers, patients with very high titers had significantly worse outcomes (p=0.02), whereas moderate elevations did not show statistical significance (p=0.25). The presence of multiple antibody positivity (≥2 markers) was also significantly associated with poor functional outcome (p=0.009). These findings indicate that systemic inflammatory burden and autoantibody profile have important prognostic implications in CNSV secondary to autoimmune disorders (Table 9).

**Table 9:** Correlation analysis: inflammatory and serological markers vs 12-month functional outcome ANA: anti-nuclear antibody; dsDNA: double-stranded deoxyribonucleic acid; ANCA: anti-neutrophilic cytoplasmic antibody; CRP: C-reactive protein

Marker	Category	Favorable Outcome (mRS 0–2), n (%)	Poor Outcome (mRS 3–6), n (%)	OR (95% CI)	Chi-square	p-value
CRP	Normal (<10 mg/L, n=50)	35 (70%)	15 (30%)	Reference		
	High (10–30 mg/L, n=60)	30 (50%)	30 (50%)	0.43 (0.20–0.95)	4.5	0.03*
	Very High (>30 mg/L, n=40)	15 (37.5%)	25 (62.5%)	0.26 (0.11–0.61)	10.2	0.001*
Autoantibody titers (ANA/dsDNA/ANCA)	Normal (below cut-off, n=60)	40 (66.7%)	20 (33.3%)	Reference		
	High (moderate elevation, n=50)	28 (56%)	22 (44%)	0.64 (0.30–1.37)	1.2	0.25 (NS)
	Very High (≥100 IU/mL dsDNA, ≥1:640 ANA, ≥50 U/mL ANCA; n=40)	17 (42.5%)	23 (57.5%)	0.37 (0.16–0.84)	5.7	0.02*
Multiple antibody positivity	Yes (≥2 markers, n=50)	20 (40%)	30 (60%)	0.38 (0.18–0.82)	6.9	0.009*
	No (<2 markers, n=100)	65 (65%)	35 (35%)			

## Discussion

In our cohort of 150 patients with CNSV secondary to systemic autoimmune disorders, the demographic and clinical characteristics were largely consistent with prior reports. The mean age at onset was in the early 40s, and there was a slight female predominance. This finding is in line with previous studies that have consistently shown a mid-life onset and female preponderance in autoimmune-related CNSV [7]. Some studies, particularly those including primary CNSV or pediatric subsets, have reported wider age distributions and younger presentation [8]. Our exclusion of pediatric patients may partly account for these differences. Clinically, the most common presentations in our cohort were stroke-like deficits, seizures, and encephalopathy. This aligns with earlier studies where focal neurological deficits and ischemic events represented the bulk of initial manifestations [7,8]. However, the proportion of seizure presentations in our patients was somewhat higher than the 20-30% typically reported [8]. This may reflect either more severe disease or referral bias in a tertiary-care setting. Encephalopathy was also relatively frequent, comparable to observations in other series of secondary CNSV [9].

Laboratory investigations revealed elevated erythrocyte sedimentation rate (ESR) and CRP in a large proportion of patients. Previous work has established inflammatory markers as supportive but non-specific indicators of vasculitic activity [10]. The frequency of ANA, anti-dsDNA, and ANCA positivity in our series was similar to systemic autoimmune cohorts, although the rate of antiphospholipid antibody positivity appeared higher than reported in earlier articles [11]. We also examined rheumatoid factor and anti-CCP antibodies in the subset of patients with underlying rheumatoid arthritis, which is not consistently described in older cohorts. These additions provide a more comprehensive immunological profile and highlight the heterogeneity of systemic disease backgrounds leading to CNS involvement. The analysis of antibody titers provided novel insights. Patients with very high titers of ANA, anti-dsDNA, or ANCA had significantly worse outcomes, while those with moderate elevations did not differ significantly from patients with negative results. This threshold effect suggests that only marked serological activity translates into meaningful clinical impact. Most previous studies have reported positivity versus negativity without titer stratification, making our categorization into normal, high, and very high a unique contribution. Furthermore, patients with multiple antibody positivity (≥2 markers) had significantly worse outcomes, consistent with the concept that broader autoimmunity reflects greater systemic disease burden and poorer neurological prognosis [12].

Radiological evaluation revealed that multiple-territory infarcts, large-vessel ischemic strokes, small-vessel lacunes, and white matter hyperintensities were frequent. Vessel wall enhancement on contrast MRI was also common. Previous studies have shown that vessel wall imaging is increasingly sensitive in detecting vasculitic changes and correlates with disease activity [9,10]. Our findings confirm that concentric vessel wall enhancement is a frequent marker in autoimmune CNSV. Compared to earlier reports where non-specific or even normal MRI findings were seen in a significant proportion of patients [7], our cohort demonstrated abnormalities in the majority. Only about 10% had a normal MRI at presentation, which is lower than previously published rates. This may be due to improved access to advanced imaging or delayed presentation with a more established disease. An important observation was the overlap of ischemic and hemorrhagic features within the same patient, underscoring the multifactorial vascular injury that characterizes CNSV. Previous studies have described similar overlap, with small- and large-vessel involvement often coexisting [8]. The relatively high rates of white matter hyperintensities may also reflect chronic inflammation and microvascular injury, findings consistent with systemic autoimmune pathology.

The correlation between laboratory and radiological features strengthens the prognostic interpretation. Patients with high CRP and very high antibody titers more frequently demonstrated severe imaging abnormalities, such as large-territory infarcts and vessel wall enhancement. This mirrors findings in earlier studies where inflammatory burden was linked with radiological severity and worse clinical outcomes [10,13]. At the same time, our observation that moderate antibody elevations were not predictive of poor outcomes highlights the importance of avoiding over-interpretation of borderline serological results. From a theoretical perspective, the threshold effect seen in serological titers suggests that the immune system must surpass a certain level of activity before clinical and radiological damage becomes apparent. This may explain why patients with mild antibody elevations can remain stable or respond well to therapy, whereas those with very high titers or multiple antibody positivity suffer aggressive disease courses. Similarly, the correlation between vessel wall enhancement and ischemic burden supports the view that direct inflammatory injury to vessel walls underlies many of the neurological manifestations of CNSV.

Overall, our demographic, clinical, laboratory, and imaging findings align with much of the prior literature but with notable differences. Higher seizure frequency, greater antiphospholipid positivity, and lower rates of normal MRI distinguish our cohort. These variations may reflect referral bias, differences in diagnostic thresholds, or more widespread use of advanced imaging techniques. The treatment strategies in our cohort largely mirrored accepted practice for systemic autoimmune vasculitis with CNS involvement, where corticosteroid induction forms the basis and further immunosuppression is guided by severity. All patients received high-dose intravenous methylprednisolone pulses followed by tapering oral steroids [14]. However, the majority required escalation with additional agents: cyclophosphamide in 26.7%, azathioprine in 16.7%, mycophenolate in 13.3%, and biologics such as rituximab or belimumab in 20%. Smaller proportions were maintained on steroids alone (13.3%) or managed supportively (10%). This distribution reflects the increasing emphasis on combination therapy rather than steroid monotherapy, consistent with contemporary reports of CNSV [15]. The relatively high use of biologics in our cohort may also signal a shift in availability and clinical confidence compared to earlier literature.

A key finding was the strong association between early initiation of immunosuppressive therapy and improved outcomes. Patients started on treatment within seven days of symptom onset were almost three times more likely to achieve a favorable functional status at one year compared with those treated later. This observation is consistent with previous reports highlighting diagnostic delay as a critical determinant of poor prognosis in vasculitis [16]. It emphasizes the clinical importance of maintaining vigilance for CNS involvement in systemic autoimmune disease and the need for rapid initiation of immunosuppression once alternative diagnoses are excluded. When outcomes were examined across treatment regimens, patients who received steroids in combination with cyclophosphamide or biologics had better recovery and lower mortality compared with those on steroids alone. These results align with multicenter studies showing rituximab to be as effective as cyclophosphamide for induction and superior for relapse prevention in some subgroups [17]. Conventional agents such as azathioprine and mycophenolate were effective in maintenance but less potent in induction. The implication is that escalation beyond steroids is necessary for most patients, and that biologics may have an increasing role in severe or refractory disease.

Relapse and recurrent admission rates further illustrate the chronic and relapsing nature of CNSV. By one year, 20% of patients had experienced relapse and 16.7% had recurrent admissions, rates that are broadly comparable with systemic vasculitis cohorts [18]. Relapse was more frequent among patients with delayed treatment initiation and those with multiple antibody positivity, suggesting that both timeliness of therapy and immunological burden influence long-term stability. These findings underscore the need for careful post-discharge monitoring, particularly in high-risk groups. Mortality in our cohort increased gradually from 10% at discharge to 13.3% at one year. While this is within previously reported ranges for CNSV, it highlights the persistent lethality of the disease despite modern therapy [19]. The incremental mortality beyond the acute phase suggests that long-term surveillance is essential, as deaths were frequently associated with relapses or systemic flares. Compared to older studies where mortality was substantially higher, our lower figures may reflect earlier recognition, better supportive care, and greater availability of advanced immunosuppressive therapy.

Laboratory and radiological correlates added further context. Elevated CRP showed a stepwise association with unfavorable outcomes, while very high antibody titers, but not moderate elevations, were significantly linked to poor prognosis. Multiple antibody positivity also strongly predicted worse outcomes, supporting the concept that cumulative autoimmune activation drives disease severity [19]. Radiologically, vessel wall enhancement, multifocal infarcts, and combined ischemic-hemorrhagic lesions were more often associated with poor outcomes and relapses, in agreement with previous studies identifying vessel wall imaging as a marker of active disease [20]. These findings suggest that combining serological and imaging markers may help define a high-risk subset requiring early aggressive therapy. Importantly, our therapeutic approach remained consistent with European League Against Rheumatism (EULAR) recommendations, which advocate corticosteroids for induction, escalation with cyclophosphamide or rituximab in severe disease, and maintenance with azathioprine or mycophenolate [21]. The alignment of our results with these recommendations strengthens the external validity of our findings. At the same time, the persistence of relapses despite therapy emphasizes the need for individualized regimens and future exploration of novel agents.

Limitations

Our study has several limitations. First, its retrospective design introduces potential biases, including variability in physician decision-making, treatment accessibility, and data completeness. Second, subgroup sizes, particularly for patients treated with specific biologics or with very high antibody titers, were small, limiting the precision of statistical estimates. Third, confounding by indication cannot be excluded, as patients receiving more aggressive regimens may have had more severe disease at baseline. Despite adjustment for key variables, residual confounding by indication cannot be excluded, as patients receiving combination or biologic therapy often had more severe disease at baseline. Fourth, although we followed patients for one year, longer-term outcomes beyond this horizon remain unknown. Finally, the study was conducted at a single tertiary-care center, which may limit generalizability to community or resource-limited settings.

## Conclusions

This study highlights the complex clinical spectrum and significant burden of CNVS secondary to systemic autoimmune disorders. Stroke-like presentations, seizures, and encephalopathy were the most frequent manifestations, and radiological features were often multifocal, reflecting widespread vascular injury. Laboratory analyses demonstrated that very high antibody titers, multiple antibody positivity, and elevated CRP were associated with poorer functional outcomes, underscoring the prognostic value of combined serological and inflammatory markers. Importantly, treatment outcomes were strongly influenced by the timing and intensity of immunosuppression. Patients receiving early therapy, particularly with steroids plus immunosuppressants or biologics, achieved better recovery and reduced relapse rates, whereas delayed therapy and steroid monotherapy were associated with poorer prognosis. Despite contemporary treatment aligned with international guidelines, mortality and relapse remained considerable. These findings emphasize the need for early recognition, aggressive individualized therapy, and long-term follow-up to improve outcomes in this challenging condition.
